# Towards characterizing the regional cerebral perfusion in evaluating the severity of major depression disorder with SPECT/CT

**DOI:** 10.1186/s12888-018-1654-6

**Published:** 2018-03-21

**Authors:** Jinming Li, Yuan Yang, Yuankai Zhu, Liqiang Zhou, Yunfeng Han, Tao Yin, Zhaoting Cheng, Guopeng Zhang, Yanxia Shen, Jing Chen

**Affiliations:** 1 Department of Nuclear Medicine, Tongji Hospital, Tongji Medical College, Huazhong University of Science and Technology, Wuhan, Hubei Province 430030 China; 2 Department of Nuclear Medicine, Renmin Hospital of Wuhan University, Wuhan, Hubei Province 430060 China; 3 Department of Neurology, Tongji Hospital, Tongji Medical College, Huazhong University of Science and Technology, Wuhan, Hubei Province 430030 China

**Keywords:** Major depressive disorder, Cerebral blood flow, SPECT/CT, [^99m^Tc]ECD

## Abstract

**Background:**

Major depressive disorder (MDD) is a common mental disorder worldwide, but now there is a lack of clinically effective assessment and management of MDD. In this study, we used technetium-99 m ethylcysteinate dimer ([^99m^Tc]ECD) SPECT/CT to characterize the regional cerebral blood flow (rCBF) status of MDD patients, and to explore an objective image assessment model of MDD which is non- or minimally-invasive, convenient and accurate in a clinical setting.

**Methods:**

The severity of MDD was assessed by three trained psychiatrists, based on scores obtained from HAMD and HAMA. [^99m^Tc]ECD rCBF SPECT/CT was performed in 20 healthy controls and 74 unipolar MDD patients before receiving the treatment. The CT attenuation-corrected SPECT images data were automatically registered, analyzed simultaneously by 3D-SSP and eZIS.

**Results:**

The mean score of HAMD and HAMA in the MDD patients was 25.49 ± 6.00, and 23.12 ± 5.83, respectively. There was a positive correlation between two scores. The MDD women had higher HAMD scores than MDD men. The decreased rCBF of MDD patients in frontal lobes (bilateral B11, B47 and right B4, B6, B10, B46), temporal lobe (right B21, B41, B42) and cingulated cortex (bilateral B24, B33), while their increased rCBF in occipital lobe (bilateral B17, B19 and left B18). Additionally, the depression severity was negatively correlated with decreased rCBF in left ventral anterior cingulate cortex B24, and was positively correlated with decreased rCBF in left inferior prefrontal gyrus B47 and increased rCBF in right associative visual cortex B19. The anxiety severity was negatively correlated with decreased rCBF in left subgenual cortex B25.

**Conclusions:**

Although the mechanism underlying the correlation is not yet fully understood, our findings indicated that the rCBF SPECT/CT may provide an objective assessment for MDD severity. It might be used monitoring therapeutic efficacy in the management of MDD.

## Background

Major depressive disorder (MDD) is a common mental disorder worldwide. It is characterized by a persistent low mood and a loss of interest or pleasure [1]. MDD is also a disabling condition that adversely affects the sufferers’ whole lives. Moreover, MDD can lead to suicide. Over 800,000 people die by suicide every year, and most of them are suffering from MDD [2]. However, fewer than 10% of MDD sufferers have ever received effective treatments, and one of the most significant barriers is lack of effective assessment and management of MDD [2].

Today, the most widely used criterion for assessing MDD is the fourth edition of the Diagnostic and Statistical Manual of Mental Disorders (DSM-IV) published by American Psychiatric Association, and the 10th revision of International Statistical Classification of Diseases and Related Health Problems (ICD-10) list by the World Health Organization. DSM-IV is more popular among mental health professionals [3]. The Hamilton Rating Scale for Depression (HAMD) is a multiple-item scale for measuring the severity of depression. However, it has been criticized for its subjectivity [4]. There is a demand for objective assessment of MDD, which is non- or minimal-invasive, convenient and accurate in a clinical setting.

Since the late 1970s, functional neuroimaging, including single photon emission computed tomography (SPECT), positron emission tomography (PET), magnetic resonance imaging (MRI) and fusing imaging (SPECT/CT, PET/CT or PET/MRI) has been extensively used in MDD [5–9]. Regional cerebral blood flow (rCBF) SPECT is a nuclear medicine imaging method in routine clinical practice, and is also one of the earliest functional neuroimaging studies in MDD [10]. According to previous research, there is almost complete agreement that blood flow in the frontal lobe is reduced in MDD patients [9, 11–14], but the controversies still exist in the extent of the rCBF involvement [15–17]. It has been speculated that the various patterns of rCBF abnormalities in MDD patients may be related to the various clinical presentation of MDD [15–17].

In this study, the rCBF status of MDD patients was characterized with technetium-99 m ethylcysteinate dimer ([^99m^Tc]ECD) SPECT/CT. The localization of abnormal rCBF was represented by Brodmann areas. In addition, we explore the relationship in MDD patients between the imaging features, clinical presentation and the severity of the symptoms. It is expected that the conventional nuclear medicine imaging could help add an objective image assessment model of MDD in current psychiatric practice that relies mainly on self-reporting of symptoms and clinical interviews.

## Methods

### Subjects

74 patients (21 men and 53 women) who fulfilled the DSM-IV criteria ^1^ for unipolar MDD were enrolled in this study, with age ranging from 20 to 72 years (mean 41.9 years old). All patients were outpatients from the department of psychiatry, Tongji Hospital of Tongji Medical College, Huazhong University of Science and Technology (HUST). The severity of MDD was assessed by three trained psychiatrists, based on scores obtained from HAMD and Hamilton Rating Scale for Anxiety (HAMA). 20 healthy controls (8 men and 12 women) were recruited through local advertisements, ranging from ages 22 to 59 (mean 38.0 years old). All MDD patients and healthy controls subjects were right handed, had never taken psychotropic medication, and had no central nervous system disorders and other medical disorders clearly affecting cerebral blood flow and cognitive function.

This study was approved by the local ethics committee at Tongji Hospital of Tongji Medical College, HUST (Permit Number: TJ-C20141220). All subjects received a detailed description of the study and gave written informed consent.

### SPECT/CT

^99^Mo-^99m^Tc generator was provided by Beijing Atomic High-tech Co., Ltd., China. The ECD kit was purchased from Jiangsu Atom Medicine Research Institute Jiangyuan Pharmaceutical Factory, China. The radiochemical purity of ^99m^Tc]ECD was over 95%.

[^99m^Tc]ECD rCBF SPECT/CT was performed using a hybrid system (Discovery NM/670, General Electric) at the department of Nuclear Medicine, Tongji Hospital of Tongji Medical College, HUST, in 20 healthy controls and 74 unipolar MDD patients before receiving treatment. The hybrid system consists of a double-headed SPECT equipped with low-energy high-resolution collimators and a 16-detector row CT. The SPECT resolution is 9.9 mm. All subjects were administrated 740 MBq (20 mCi) [^99m^Tc]ECD after resting 30 min with eyes closed in a dimly quiet room. Followed resting another 30 min, SPECT/CT fusing imaging were performed with a 20% window width centered at 140 KeV and a 128 × 128 matrix. Reconstructed images were displayed as CT images, SPECT images, and fusion images in the axial, coronal, and sagittal planes as well as maximum intensity projection images.

### Image analysis

The images were interpreted by three nuclear medicine physicians. The abnormal rCBF was defined using a three-dimensional stereotactic surface projection (3D-SSP) technique, developed as part of a neurologic statistical image analysis (NEUROSTAT) program (Department of Internal Medicine, University of Michigan, Ann Arbor, MI, USA). All CT attenuation-corrected SPECT data were automatically registered, analyzed and compared with a normalized database using 3D-SSP. A Z-score-based semi-quantitative analysis was used, and the Z-score of 1.5 was set as a cutoff point to identify the brain areas with abnormal rCBF. The localization of abnormal rCBF was presented by Brodmann areas (B). Besides, regions of interest (ROIs) were drawn on the transverse sections of the SPECT/CT fusion images by one operator over caudate nucleus, globus pallidus, hippocampus and cerebellum, which cannot be identified clearly by B. The mean counts of cerebellum were measured correcting for background counts, which mostly remains unchanged rCBF in all subjects according to our observational study. The fraction of mean counts in the targeted area ROI-to-cerebellum ROI was calculated as [^99m^Tc]ECD uptake index (UI).

The same CT attenuation-corrected SPECT images data were also processed using statistical parametric mapping (SPM12; Functional Imaging Laboratory, University College London, London, UK) and easy Z score imaging system (eZIS; Fijifilm Ri Pharma Co., Kanazawa, Japan).

### Statistical analysis

Data were processed using the IBM SPSS Statistics V19.0 (SPSS Inc., Chicago, IL, USA). Results were expressed as mean ± SD. A *P* value (*P*<0.05) is considered statistically significant. Student t-test was used to compare the difference in the HAMD score / HAMA score between groups, and to identify abnormal rCBF between healthy controls and MDD patients. Pearson correlation analysis and multiple linear regression analysis were used to infer causal relationships between regional cerebral Z-scores and HAMD scores / HAMA scores in MDD patients. The result of eZIS was visualized using xjView toolbox (http://www.alivelearn.net/xjview).

## Results

**Fig. 1:**
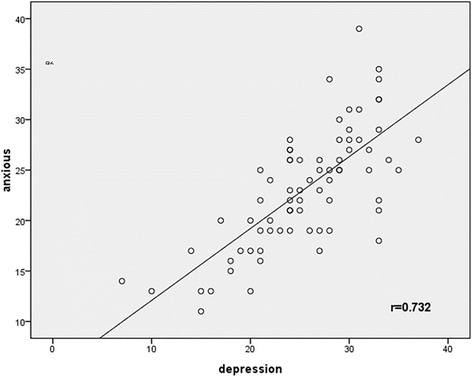
The correlation between the score for HAMD and that for HAMA

The mean HAMD score and the mean HAMA score in the MDD patients were 25.49 ± 6.00 and 23.12 ± 5.83, respectively, and as for healthy controls, the mean scores were 2.70 ± 1.42 and 0.65 ± 0.88, respectively. There was a positive correlation between two scores for MDD patients (*r* = 0.732, *p* = 0.000) (Fig. 1).

It is still controversial whether there are correlations between the MDD severity and the patient characteristics. In this study, the HAMD score showed no correlations with the ages of MDD patients (*r* = 0.134, *p* = 0.255). The MDD women (26.66 ± 5.11) had higher HAMD scores than the MDD men (22.52 ± 7.08, *t* = 2.801, *p* = 0.007).

Compared with the normal controls data, 3D-SSP analysis showed that the MDD patients had decreased rCBF in frontal lobe (bilateral B4, B9, B10, B11, B47 and left B8 and right B6, B44, B46), parietal lobe (left B312), temporal lobe (right B21, B41, B42), cingulated cortex (bilateral B24, B32) and hypophysis. And it also showed in the MDD patients increased rCBF in parietal lobe (right B7), temporal lobe (right B28), occipital lobe (bilateral B17, B19 and left B18) and cingulated cortex (left B23 and right B31, B33). The detailed Z-scores were shown in Table 1. There was no statistically significant difference between MDD patients and normal controls for rCBF in hippocampus, caudate nucleus and globus pallidus (Table 2).

**Table 1:** Z-scores of the increased/decreased rCBF areas in MDD patients from 3D-SSP

			Z-score		t value	*p* value
			MDD	CON		
INC:	B7	R	4.60 ± 1.78	3.74 ± 1.97	2.371	0.019
	B17	L	6.12 ± 1.22	4.80 ± 1.76	4.703	0.000
		R	5.47 ± 1.70	4.30 ± 1.45	3.685	0.000
	B18	L	6.35 ± 0.96	5.72 ± 0.99	3.323	0.001
	B19	L	6.70 ± 0.80	6.38 ± 0.80	2.033	0.044
		R	6.19 ± 1.08	5.41 ± 1.13	3.593	0.000
	B23	L	3.43 ± 1.92	2.67 ± 1.84	2.050	0.043
	B28	R	1.60 ± 2.83	0.00 ± 0.00	4.873	0.000
	B31	R	4.52 ± 1.96	3.59 ± 2.00	2.414	0.017
	B33	R	3.36 ± 2.53	2.49 ± 1.89	2.086	0.039
DEC:	B123	L	1.79 ± 2.28	2.77 ± 1.93	2.312	0.023
	B4	L	1.46 ± 1.99	2.39 ± 2.09	2.354	0.020
		R	6.39 ± 1.09	6.77 ± 0.37	2.658	0.009
	B6	R	2.17 ± 2.05	3.18 ± 1.82	2.590	0.011
	B8	L	−3.84 ± 1.97	−2.99 ± 1.75	2.276	0.025
	B9	L	1.72 ± 1.61	2.69 ± 1.17	3.653	0.000
		R	1.79 ± 1.67	2.74 ± 1.58	2.941	0.004
	B10	L	1.55 ± 1.70	2.73 ± 1.78	3.466	0.001
		R	1.33 ± 1.77	2.91 ± 1.58	4.723	0.000
	B11	L	2.50 ± 2.15	3.41 ± 1.86	2.357	0.021
		R	2.81 ± 2.16	3.74 ± 1.94	2.256	0.026
	B21	R	2.89 ± 1.88	3.70 ± 1.88	2.205	0.030
	B24	L	1.61 ± 1.84	2.26 ± 1.32	2.162	0.033
		R	3.60 ± 1.64	4.18 ± 0.91	2.436	0.016
	B32	L	1.72 ± 1.61	2.95 ± 1.31	4.398	0.000
		R	1.72 ± 1.66	3.31 ± 1.25	5.772	0.000
	B41	R	1.45 ± 1.86	2.30 ± 2.01	2.260	0.026
	B42	R	1.67 ± 1.91	2.46 ± 1.87	2.115	0.037
	B44	R	2.25 ± 1.92	3.22 ± 1.65	2.817	0.006
	B46	R	1.96 ± 1.99	3.02 ± 1.94	2.739	0.007
	B47	L	1.90 ± 1.88	2.92 ± 1.21	3.515	0.001
		R	2.46 ± 1.84	3.46 ± 1.29	3.392	0.001
	hypophysis		6.84 ± 0.45	7.00 ± 0.00	3.109	0.003

*L* left, *R* right, *DEC* the decreased rCBF areas, *INC* the increased rCBF areas, *rCBF* regional cerebral blood flow, *MDD* major depressive disorder, *CON* control

**Table 2:** Quantitative assessment of rCBF changes in caudate nucleus, globus pallidus and hippocampus from regions of interest method

		UI (mean ± SD)		t value	*p* value
		MDD group	Control group		
HIP	L	0.698 ± 0.103	0.718 ± 0.087	0.789	0.435
	R	0.677 ± 0.074	0.667 ± 0.088	0.527	0.600
CAU	L	0.850 ± 0.110	0.854 ± 0.117	0.141	0.888
	R	0.799 ± 0.102	0.800 ± 0.096	0.043	0.966
GLO	L	0.928 ± 0.110	0.967 ± 0.129	1.361	0.177
	R	0.910 ± 0.117	0.957 ± 0.099	1.657	0.101

*MDD* Major depressive disorder, *HIP* Hippocampal gyrus, *CAU* Caudate nucleus, *GLO* Globus pallidus, *UI* Uptake index, *rCBF* Regional cerebral blood flow

The HAMD score had a negative correlation with decreased rCBF in left ventral anterior cingulate cortex B24, a positive correlation with decreased CBF in left inferior prefrontal gyrus B47 and increased rCBF in right associative visual cortex B19 (*R* = 0.400, F = 4.453, *p* = 0.006). In addition, there was a negative correlation between the HAMA score and decreased rCBF in left subgenual cortex B25 (*r* = − 0.309, *p* = 0.007).

**Fig. 2:**
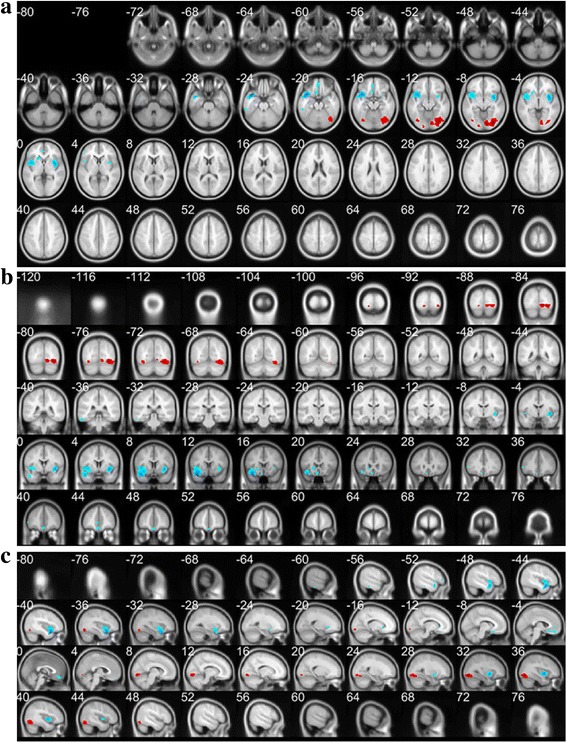
The decreased (blue) and increased (red) rCBF foci in the reconstructed transverse slices (a), coronal slices (b) and sagittal slices (c) of MDD patients, which was analyzed by SPM and eZIP and viewed by xjView toolbox

The differences of rCBF between MDD patients and normal controls were significant at the cluster lever (*P*_FWE-corr_ < 0.05) in the SPM and eZIP analysis. The results were listed in Table 3, and showed in Fig. 2 which was drawn from xjView toolbox and showed the decreased and increased rCBF foci in MDD patients.

**Table 3:** Talairach Coordinates and Z-scores of areas with rCBF changes in MDD patients

Number of clusters	Co-ordinates			Region	Z-score (*P*_FWE-corr_ < 0.05)
	X	Y	Z		
INC: 2	**-32**	**-80**	**-10**	left occipital lobe (B17、B18、B19、B37)	5.91
	-8	-82	-8		5.43
	**36**	**-70**	**-14**	right occipital lobe (B17、B18、B19、B37)	4.87
DEC:4	**38**	**14**	**-24**	right temporal lobe (B22、B38、B41、B42), right insula and right frontal lobe (B11、B44、B45、B47)	6.07
	42	16	-12		5.30
	46	6	-24		5.09
	**-32**	**4**	**-4**	left insula, left frontal lobe (B11、B44、B45、B47), left temporal lobe (B22、B38、B41、B42) and ^a^left anterior cingulate cortex (B24、B33)	5.30
	-38	-2	-2		5.07
	**20**	**22**	**0**	right frontal lobe (B4、B6、B10、B11、B44、B45、B46、B47), right temporal lobe (B21、B22、B38、B41、B42), right insula and ^a^right anterior cingulate cortex (B24、B33)	4.50
	**8**	**22**	**-20**	right frontal lobe (B4、B6、B10、B46)	4.40

*L* Left, *R* Right, *DEC* The decreased rCBF areas, *INC* the increased rCBF areas, *rCBF* Regional cerebral blood flow, *PFWE-corr p*-value with FEW correction in %, FEW family-wise error

^a^ only exist in *P* < 0.001

According to 3D-SSP and eZIP analysis, the consistent results showed the decreased rCBF of MDD patients in frontal lobes (bilateral B11, B47 and right B4, B6, B10, B46), temporal lobe (right B21, B41, B42) and cingulated cortex (bilateral B24, B33), while their increased rCBF in occipital lobe (bilateral B17, B19 and left B18).

## Discussion

MDD is a heterogeneous disorder with various clinical presentation and inconsistent response to treatment. But its pathogenesis is inadequately understood [6, 18]. The mechanism that underlies the altered rCBF in MDD patients is not fully elucidated, however, previous studies [19, 20] suggested that the depressive symptoms were associated with reduced parasympathetic and increased sympathetic tone resulting in a broad range of hemodynamic changes, including elevated systemic vascular resistance. In addition, activation of immune-inflammatory pathways in MDD may also contribute to the process. Our study showed that more severe MDD patients would experience more anxiety. It has been suggested to be caused partly by brain chemistry neurotransmitter imbalances [21]. Antidepressant medication and behavioral therapy can help MDD patients overcome both depression and anxiety. However, clinicians have observed when depression occurs together with anxiety, symptoms are more severe than when depression or anxiety occurs alone, furthermore, it may be likely to develop treatment-resistant depression [22]. Additionally, our study also showed that the severity of depression had no correlations with age, and that women were found more likely than men to develop severe depression, the possible reasons may be as follows: women are more likely than men to show mood amplification; women have a greater propensity to be more passive than men; women may be exposed to more stressful life events than men [23].

To minimize the deviation errors caused by analysis software packages, 3D-SSP and eZIP image-processing programs were applied in the same CT attenuation-corrected SPECT images data of MDD patients and normal controls. According to previous studies [24, 25], the difference between 3D-SSP and eZIP lies in smoothing process after anatomic standardization. The smoothing process by eZIP is based on an SPM algorithm and effectively enhances the signal-noise ratio of images, which might overestimate cerebral hypoperfusion [24, 25]. So we apply corrections for multiple comparisons based on family-wise error (FEW), and *P*_FWE-corr_ < 0.05 is considered statistically significant in eZIP analysis. On the other hand, due to uninvolving such a smoothing process the preanalysis image reconstruction conditions might affect the results of 3D-SSP. Therefore, in 3D-SSP analysis all CT attenuation-corrected SPECT images data of MDD patients were compared with a normalized database and those of normal controls data simultaneously. Finally, the consistent results from 2 statistical image analysis methods were defined as the abnormal rCBF status of MDD patients. By 3D-SSP and eZIP analysis, we concluded that the decreased rCBF of MDD patients was in frontal lobes (bilateral B11, B47 and right B4, B6, B10, B46), temporal lobe (right B21, B41, B42) and cingulated cortex (bilateral B24, B33), while their increased rCBF was in occipital lobe (bilateral B17, B19 and left B18). Thus it can be seen that the rCBF abnormalities of MDD patients appear in bilateral cerebral hemisphere, and the hypoperfused regions showed right-hemisphere dominance while the area of hyperperfusion had a slight left-hemisphere dominance.

In the previous studies about brain structure [7, 26], metabolism [27] and perfusion [17], the medial prefrontal cortex (mPFC) and anterior cingulate cortex (ACC) in MDD patients have been found increased functional connectivity, which was related to one of possible pathophysiological mechanisms of depression——dysfunction of default mode network (DMN) [21, 28]. The hypoperfused areas of frontal lobes and cingulated cortex in MDD patients of this study largely coincided with what have been reported in the past [9, 16, 29, 30]. Although there was no consensus in regard to the hypoperfusion of temporal lobe and the hyperperfusion of occipital lobe, some researchers [9] have mentioned the abnormal perfusion of these cerebral regions in patients with treatment-resistant depression, and other researchers [31] suggested that abnormal activation of the occipital lobes may be an initiating factor cognitive disorder in depressed patients. We found there was no significant difference in the cerebellar blood flow of MDD patients as compared with that of normal controls, which was inconsistent with the findings of Gardner et al^28^, in which cerebellum resulted to be heavily implicated in a large cohort of patients with persistent depressive disorder (PDD). The discrepancy may be due to the various clinical presentations of MDD patients, and interindividual variability in the patterns of functional connections between brain regions also existed in the specific MDD symptom clusters [17, 28]. The MDD patients involved in our study mostly have high HAMD scores and HAMA scores with the most common complaints——feeling down and insomnia, and this may be the reason why the different distribution of abnormal rCBF appeared in the MDD patients of this study.

In addition, our present study revealed that among these recruited MDD patients with relatively high HAMD and HAMA scores, the depression severity was negatively correlated with decreased rCBF in left ventral anterior cingulate cortex B24, and was positively correlated with decreased rCBF in left inferior prefrontal gyrus B47 and increased rCBF in right associative visual cortex B19. It also indicated that the anxiety severity was negatively correlated with decreased rCBF in left subgenual cortex B25. These results may be helpful in monitoring therapeutic efficacy of MDD, although a large-scale study is still necessary to verify the above characteristics of rCBF alteration in the progression of MDD and during follow-up after treatment. On the other hand, there were several additional limitations in this study including: our results weren’t compared with the findings from MRI techniques, such as 3D-arterial spin labelling; the age range of the included healthy controls (22–59 years) is smaller than that of MDD patients (20–72 years). Further studies are required to resolve these issues.

## Conclusions

In conclusion, blood flow abnormalities were found in bilateral brain hemisphere of MDD patients with right-hemisphere dominant hypoperfused regions and a slight left- hemisphere dominance in the area of hyperperfusion. The depression severity was associated with decreased rCBF in B24 and B47 of left hemisphere, and increased rCBF of right B19. The rCBF SPECT/CT may provide an objective assessment for MDD severity. And it might be used monitoring therapeutic efficacy in the management of MDD.

## Abbreviations

[^99m^Tc]ECD: [^99m^Tc]ethylcysteinate dimer
3D-SSP: Three-dimensional stereotactic surface projection
ACC: Anterior cingulate cortex
B: Brodmann areas
CT: Computed tomography
DMN: Default mode network
DSM-IV: The fourth edition of the Diagnostic and Statistical Manual of Mental Disorders
eZIS: easy Z score imaging system
HAMA: Hamilton Rating Scale for Anxiety
HAMD: Hamilton Rating Scale for Depression
HUST: Huazhong University of Science and Technology
ICD-10: The 10th revision of International Statistical Classification of Diseases and Related Health Problems
MDD: Major depressive disorder
mPFC: Medial prefrontal cortex
MRI: Magnetic resonance imaging
PET: Positron emission tomography
rCBF: Regional cerebral blood flow
ROIs: Regions of interest
SPECT: Single photon emission computed tomography
UI: Uptake index
